# Migration of Latin American nurses to Spain 2006–2016: a case study

**DOI:** 10.1111/inr.12511

**Published:** 2019-04-16

**Authors:** M. Pastor‐Bravo, S. Nelson

**Affiliations:** ^1^ Lawrence S. Bloomberg Faculty of Nursing University of Toronto Toronto ON Canada; ^2^ Department of Nursing Universidad de Murcia Cartagena, Murcia Spain; ^3^ University of Toronto Toronto ON Canada

**Keywords:** Case Study, International Nurses, Latin America, Migrant Nurses, Migration, Nursing, Spain

## Abstract

**Aim:**

To examine the migration of nurses from Latin America to Spain over the period from 2006 to 2016.

**Background:**

This study examines the impact of the 2008 global economic crisis on migration flows of nurses to Spain from its major source countries of Latin America.

**Methods:**

Using an exploratory case study, we present original data provided by the Ministry of Education, Culture and Sport of the Government of Spain upon request on applications and success rates for credential recognition of nurses intending to immigrate to Spain, with an extended analysis of Latin American applications which account for the 70% of skilled worker migration to Spain.

**Results:**

Successful applications for credential recognition of overseas nursing qualifications plummeted from a peak of 1384 in 2007 to 55 in 2016. Migration intentionality also decreased but has undergone a slight increase in recent years.

**Discussion/Conclusion:**

We found that the economic crisis effectively closed the door to internationally educated nurses to work as nurses in Spain. Moreover, the denial of official recognition of nursing credentials appears to be unaffected by the existence of bilateral trade and mobility agreements between Spain and source countries. We conclude that the level of nursing migration to Spain is a sensitive indicator of domestic labour market conditions.

**Implications for health policy:**

Despite the lack of any transparent policy on the credential approvals, in practice the government is limiting access to the nursing labour market by overseas education nurses. We urge that attention be paid by health human resource planners on the intersection between labour market and migration trends to support a transparent and data‐informed discussion by all stakeholders on the current state of the nursing labour market in Spain and its future needs.

## Introduction

The global economic crisis (2008–2014) had an effect on the migration trends of nurses to Spain. On one hand, there was an increase in the migration of Spanish nurses out of Spain from 2009 to 2014 (Galbany‐Estragués & Nelson 2016). More than 9200 Spanish nurses were working in other European countries in 2014 (OECD 2017). In fact, Spain and Portugal are the major suppliers of nurses to Great Britain and Germany (Marc et al. 2018). While these migrations represent a loss of human capital, the country also benefits when nurses migrate to Spain (Hernandez et al. 2016). This migration occurs typically from Latin American countries (Sanabria Mora 2011).

Our objective was to examine the migration of nurses from Latin America to Spain over the course of the decade from 2006 to 2016. We also explored the sensitivity of nurse migration flows to both the economic conditions in Spain and the existence of mobility agreements between Spain and source countries.

Working from a comprehensive data set of nurses who immigrated to Spain from Latin American countries between 2006 and 2016, we undertook a retrospective analysis that examined the little‐understood phenomenon of nursing migration flows between Spain and Latin America. This paper presents original unpublished data on immigrants with nursing qualifications over this period of time.

## Literature review

### Spanish migratory, sociopolitical and health system context

Until the late 1990s, few nurses migrated to Spain. High levels of unemployment, comparatively low salaries, instability at work, temporary work and lack of social recognition made Spain an unattractive destination compared with other European countries (González López‐Valcárcel et al. 2011). In fact, prior to the 1990s, Spain was a source rather than a receiving country for migration overall. It was not until 1985 that its first immigration law was introduced (BOE 1986).

By the late 1990s, the growth of the Spanish economy made Spain a target for both legal and illegal immigration (Alonso‐Pérez & Furio Blasco 2007). In 2001, the accidental deaths of 12 Ecuadoreans revealed to the public the magnitude of Spain's problem with illegal immigration. Workers from Ecuador and Colombia, many of whom were in situations of exploitation and even danger, had been flowing into the country through illicit channels (European Parliament 2009). This prompted Spain's implementation of the Greco Program 2001–2004 to regulate and control immigration as well as to promote the return of migrants to their countries of origin (González López‐Valcárcel et al. 2011).

In 2004, a new regulation on foreigners was approved and a catalog of occupations created to satisfy the demands of the Spanish market. This regulation increased the flow of legal entries of workers hired prior to their arrival in Spain (European Parliament 2009). With regard to human resources in health, several studies report that planning had been inadequate and that Spain was dependent on the immigration of health professionals (González López‐Valcárcel et al. 2011; Hernandez et al. 2016). And that Spain's needs were primarily being met by Latin American health professionals (Hernandez et al. 2016).

However, things changed with the global economic crisis in 2008 and the subsequent adoption of ‘protectionist’ (Cerna et al. 2015) and ‘restrictive’ migration policies (OECD 2009), challenging workers’ migration rights (Huddleston & Niessen 2011; OECD 2010).

During the crisis, The Royal Decree‐Law 16/2012 was implemented to ‘guarantee the sustainability of the National Health System and improve the quality and safety of its benefits’ (BOE 2012, p. 1). This Decree‐Law affected the universality of the Spanish health system by excluding adult immigrants not registered or authorized as residents (except for emergency and obstetrical cases), among others. The transformation of the Spanish health system and the implementation of austerity measures during the crisis also slowed down the average annual growth rates in health spending per capita (OECD 2017), compromised job security for nurses and increased the number of patients per nurse, affecting quality of care (Aiken et al. 2016; Galbany‐Estragués & Nelson 2016).

## Methods

### Design

We undertook an exploratory case study design to allow for an in‐depth analysis of a relevant but little known phenomenon (Yin 2018). In this case, the evolution of Latin American nurse migration to Spain (2006–2016), within its context.

Case studies have been widely used in political science and sociology (Gomm et al. 2009). In this instance, the use of a case study will provide the mechanism to afford a clearer understanding of the nurse migration process through the sociopolitical Spanish context affecting migratory movements and the Spanish health system.

### Study population

We examined Latin American nurses’ intention to migrate to Spain by analysing the applications for official recognition of their nursing degrees by the Spanish Ministry of Education, Culture and Sport (MECS) between 2006 and 2016, which is the total period for which data are available. We further reviewed the volume and trends in applications and examined the success rate over time.

Non‐European foreign nurses need to apply for official recognition of their degree to work in Spain (MECD, SEI 2015). This process is regulated by Directive 967/2014, which states that once the official recognition of the degree is conceded, the foreign nurse has access to all the rights of the regulated profession with the same conditions as nurses who obtained their degree in Spain (MECD 2017).

We use the data on credential recognition as there are no data on skilled workers, including nurses, who migrate Spain. While degree recognition does not necessarily equate with migration, we believe it stands as a useful proxy for migration as the higher salary and better working conditions offered in Spain compared to Latin American countries means there is a reasonable likelihood of successful applicants immigrating (Sanabria Mora 2011).

The inclusion criteria for this study were as follows: (1) Latin American educated nurses, (2) applied for credential recognition to work as a nurse in Spain between 2006 and 2016.

Data for successful and unsuccessful applications for credential recognition are included.

### Data collection

In addition to data from MEC (2006–2016), we used data from the National Institute of Statistics (INE 2014–2017), and the Health Statistics Organization for Economic Cooperation and Development (OECD 2013–2016). Other data sources included policy documents, laws, international organizations or instruments to regulate labour migration, free trade agreements between the studied countries and Spain, and regulations for obtaining official recognition to practice nursing in Spain.

The numerical data consisted of applications for official recognition of nursing degrees and those granted from 2006 to 2016, disaggregated by year and country of origin. These data were provided upon request by the MECS.

### Data analysis

Numerical data were grouped into Excel tables and exported to graphics, which facilitated the identification of patterns throughout the period studied (2006–2016). To understand the factors influencing nurse migration from Latin America, we compared the quantitative data with policy trends, migratory agreements and economic and major political events that affected the various countries over this period.

## Results

### Nurse migration to Spain

**Figure 1:**
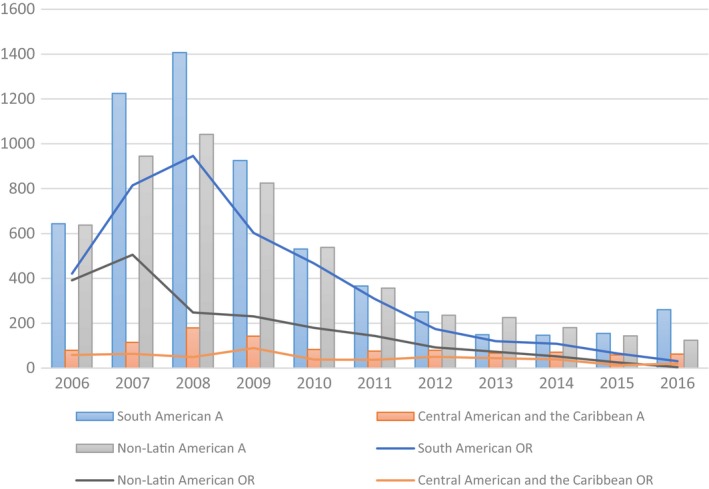
Applications for official recognition and official recognition granted in South American, Central American and Caribbean and non‐Latin American countries. Source: Data request to the Ministry of Education, Culture and Sport of the Government of Spain. A, Application (Bar); OR, Official Recognition (line).

MCES data show that from 2006 to 2016, 6505 nursing degrees were officially recognized in Spain. Of these, 4059 were granted to South American nurses and 501 to nurses from Central America with most nurses coming from South American, Central American, Mexico and Caribbean countries. Fifty‐seven per cent of the applications for credential recognition were from Latin America (see Fig. 1; Table 1).

**Table 1:** Applications for official recognition and effective official recognition of nursing degrees in Spain in Latin American countries and all the countries since 2006–2016

Year	Latin American countries			All the countries		
	OR	A	%	OR	A	%
2006	479	723	66.25	871	1360	64.04
2007	879	1344	65.40	1384	2285	60.57
2008	995	1586	62.73	1243	2628	47.30
2009	692	1067	64.85	923	1892	48.78
2010	504	614	82.08	683	1152	59.29
2011	345	441	78.23	488	799	61.07
2012	224	327	68.50	316	565	55.93
2013	164	215	76.28	237	441	53.74
2014	148	218	67.89	200	398	50.25
2015	79	213	37.09	105	357	29.41
2016	51	324	15.74	55	447	12.30
2006–2016	4560	7072	64.48	6505	12324	52.78

Source: Data request to the Ministry of Education, Culture and Sport of the Government of Spain.

%, Percentage of application granted; A, Application; OR, Official Recognition.

With respect to the success rate of applications between 2006 and 2016, the percentage of applications granted to Latin Americans fell sharply in 2015 (37.09%) and 2016 (15.74%). In these years, there was a matching decrease in the success rate of nurses from non‐Latin American countries (18.05% in 2015 and 3.22% in 2016; Table 1).

Both the total applications and the total official degree recognitions granted to Latin American applicants peaked in 2008. The total number of applications began to decline year after year until 2014, then rose again in 2016. In all, successful applications for credential recognition of overseas nursing qualifications plummeted from a peak of 1384 in 2007 to 55 in 2016 (Table 1).

### Latin American Nurse migration to Spain by country

The largest number of applicants requesting official recognition between 2006 and 2016 was from Peru and Colombia, followed by Cuba, Venezuela and Argentina, while the leading countries with the most credential recognition granted were Colombia and Peru, followed by Cuba, Venezuela and Ecuador (see Table 2).

**Table 2:** Applications for official recognition and effective official recognition of nursing degrees in Spain by country since 2006–2016

Country		06	07	08	09	10	11	12	13	14	15	16	Total 06–16	Total 06–16%
Argentina	A	71	67	78	55	37	19	7	18	11	7	12	382	51.04
	OR	39	39	38	13	24	13	7	10	4	3	5	195	
Brazil	A	10	17	18	19	12	7	8	8	10	16	7	132	54.54
	OR	5	4	13	10	8	10	7	7	2	5	1	72	
Bolivia	A	23	27	26	18	21	21	11	7	4	6	7	171	67.25
	OR	14	21	18	7	24	9	11	6	5	0	0	115	
Chile	A	18	29	32	21	12	13	20	8	14	12	13	192	59.89
	OR	13	13	20	11	25	4	7	10	5	4	3	115	
Colombia	A	159	606	617	295	159	88	31	25	23	30	61	2094	75.93
	OR	86	436	549	187	157	92	38	23	14	4	4	1590	
Ecuador	A	62	58	44	25	12	18	8	9	4	8	5	253	81.42
	OR	48	40	38	33	18	16	5	2	3	3	0	206	
Peru	A	248	343	491	413	222	154	132	55	42	28	43	2171	66.28
	OR	178	215	223	309	174	112	67	47	56	41	17	1439	
Paraguay	A	8	7	18	20	10	6	4	5	4	4	8	94	47.87
	OR	7	3	6	5	7	7	6	1	1	1	1	45	
Uruguay	A	24	29	40	13	6	3	5	1	1	2	6	130	53.07
	OR	18	16	18	7	2	3	3	1	0	1	0	69	
Venezuela	A	21	42	43	46	40	37	24	13	34	42	98	440	48.40
	OR	13	28	23	21	27	42	23	13	19	4	0	213	
Cuba	A	60	99	136	101	52	59	58	53	57	44	42	761	53.08
	OR	49	56	35	78	19	24	45	36	35	11	16	404	
El Salvador	A	1	2	12	12	3	1	1	2	1	2	3	40	40.00
	OR	1	0	0	6	4	3	2	0	0	0	0	16	
Nicaragua	A	2	1	4	8	6	0	2	0	2	1	2	28	46.42
	OR	1	0	3	0	0	3	2	2	1	1	0	13	
Dominican Republic	A	11	3	19	11	14	5	5	5	3	4	4	84	36.90
	OR	7	5	5	1	7	2	0	3	0	0	1	31	
Mexico	A	4	7	7	6	5	6	6	3	5	4	6	59	44.06
	OR	0	1	4	3	5	5	1	2	2	1	2	26	
Rest of C.A and Caribe	A	1	7	1	4	3	4	5	3	3	3	7	41	26.82
	OR	0	2	2	1	3	0	0	1	1	0	1	11	

Source: Data request to the Ministry of Education, Culture and Sport of the Government of Spain.

%, Percentage of application granted; A, Application; C.A, Central America; OR, Official Recognition.

#### South America

From 2006 to 2016, the countries with the most official recognition of their nursing degrees granted were also Colombia and Peru, followed by Venezuela, Ecuador, Argentina and Chile. Before the crisis, the highest numbers of applications and officially recognized degrees were from nurses from Colombia and Peru, followed by Ecuador, Argentina and Venezuela. However, between 2014 and 2016, Peru, Colombia, Venezuela, Argentina and Chile were the countries with the most successful applications for credential recognition, while nurses from Venezuela, Peru, Colombia, Argentina and Chile made the most requests.

Nonetheless, nurses from South America had fewer successful applications in recent years. For instance, the success rate of applications from Peru decreased by 14.89% between 2008 and 2016. For Ecuador, the decrease was 86.36% for the same period.

#### Central America, Mexico and the Caribbean

Few applications for official recognition of degrees were received from or granted to nurses from Costa Rica, Guatemala, Honduras, Panama or Haiti. The number of applications was higher in El Salvador and Nicaragua, but less than 50% of the applications were granted. Between 2006 and 2016, the most applications and the most successful applicants from Central America were Cuban nurses, followed by those from the Dominican Republic and Mexico.

## Discussion

The majority of nursing applications for official recognition come from Latin American countries. This is because language fluency is a key element in credential recognition and as such it favours Spanish‐speaking countries. Furthermore, immigrants tend to follow language‐specific pathways from French‐ or English‐speaking source countries to French‐ or English‐speaking destination countries (Adserà & Pytliková 2015; Otero 2004). In fact, to date there have been few applications for degree recognition from English‐ or French‐speaking Latin American countries.

The Spanish government's and employers’ apparent preference for Latin American immigrants for cultural and linguistic reasons (Otero 2004), along with the greater ease of workforce integration of Latin American professionals into the Spanish health system (Finotelli & Mateos 2015), resulted in applications from Spanish‐speaking Latin American nurses having a higher success rate than other applicants.

Most foreign nurses who came to Spain between 2006 and 2016 were from Colombia and Peru. Ecuador is the country with the highest percentage of applications for official recognition of degrees granted in this period. These three countries have bilateral migration agreements with Spain which are ostensibly to facilitate this migration; however, the authors did not find a consistent relationship between the existence of bilateral agreements and nursing migration flows, since the number of guaranteed applications did not increase, nor was there any evidence of preferential treatment of applications after the implementation of these agreements.

Nurses from El Salvador, Nicaragua, Costa Rica, Guatemala, Honduras or Panama follow other migratory patterns, one of which is to the United States, the most popular destination for health workers globally, and where these countries have strong political and social links (England 2015; Squires et al. 2016). The second pathway is an intra‐regional migration pattern. The facilitated recognition of credentials between these countries, and their geographic proximity, promotes migration between neighbouring countries, with Costa Rica considered a receptor country for nurses from within Central America (Cabrera Sandoval 2011; OPS 2011).

In Mexico, the official recognition of nursing qualifications (and all degrees) increased between 2008 and 2011, likely due to the 2008 project which recruited Mexican workers, but which had a limited impact due to the onset of the crisis (European Parliament 2009). In the Dominican Republic, there was an increase in applications for official recognition after 2008, which may be an effect of the agreement with Spain that was implemented in 2007. In neither Mexico nor the Dominican Republic did the mobility agreements result in higher success rates of applications from nurses.

From 2009, interest from Latin American nurses in working in Spain declined, with a resulting decrease in applications for official recognition during 2010–2013. This decline may have been due to the reduction and cessation of the recruitment of nursing staff that occurred as a result of the economic crisis (Finotelli & Mateos 2015), or a result of the increased precariousness of working conditions in Spain overall (Galbany‐Estragués & Nelson 2016).

In 2016, there was a significant increase in applications for official recognition of nursing degrees from immigrants from Latin American countries. There may be a number of factors that created this increased demand. First, publicity about the end of the crisis promulgated by the Ministry of Economy, Industry and Competitiveness (MEIC 2014a,2014b) may have incorrectly messaged a return to pre‐crisis levels of approvals. This increase in the number of nursing applications only applies to Latin American applications, which may have been influenced by worsening situations in those countries, as in the case of Venezuela, where the economic crisis has caused a drastic increase in applications for official recognition in recent years. Despite the increase in the number of nursing applications, the success rate for Latin American applications continued to fall. This may be in part due to the number of people registered as unemployed in Spain in 2016 who selected nursing as their preferred job option (5694; SEPE 2016a). In 2016, 1.44 times more nurses were registered as unemployed in Spain than in 2007 (3942; SEPE 2016b). Nonetheless, the unemployment figures were decreasing overall and the number of new contracts for nurses in the health sector has been increasing in recent years (Galbany‐Estragués & Nelson 2016; SEPE 2016b). So far, these positive changes in work availability for Spanish nurses have not affected the decline in approval for foreign credential recognition. It is not possible to determine the reason behind the continued decline in approvals from the data, and this question would benefit from further investigation.

### Limitations

We examine nursing migration through analysis of applications and success rates for the official recognition of Latin American nursing degrees, since it is an essential requirement for non‐European nurses to work in Spain (MECD, SEI 2015). While degree recognition does not equate with migration, we consider the applications and success rates for the official recognition of foreign nursing degrees as appropriate proxy data for this preliminary study.

## Conclusions

The economic crisis affected the migratory pattern of nursing in Latin America, which was increasing year by year until a sudden and continuous decline from 2009 to the present.

The decline of foreign nurses who migrated to Spain was especially severe during the period of most intense economic recession in Spain (2012–2014). However, the number of Latin American nursing degrees officially recognized has declined in Spain since 2009, and since 2008 in the case of all nursing degrees from all countries. Finotelli & Mateos (2015) and Hernandez et al. (2016) observe that nursing migration has been more affected than the migration of other health professionals.

The MECS continues to restrict the entry of Latin American nurses, granting fewer approvals year after year, although there is little public awareness of this trend. The Ministry has the discretion to award or deny official recognition of foreign credentials, and the success of applications does not appear to be consistently correlated with labour mobility agreements signed by the Spanish government. Our nursing data confirm Panizzon et al.'s (2015) general observation that mobility agreements do not lead to preferential access to the Spanish workforce.

## Implications for policy

High levels of unemployment, continued outmigration of Spanish nurses and increased casualization of the Spanish labour market in health care have led to significant changes in employment security. Despite the lack of any transparent shift in policy on credential approvals, in practice the government is severely limiting access to the labour market, apparently in response to these trends and without consideration of bilateral agreements between countries.

We urge that attention be paid by health human resource planners at national and regional levels on the intersection between labour market and migration trends to support a transparent and informed discussion by government, employer and education stakeholders on both the current state of the nursing labour market in Spain and its future needs. Improved access to comprehensive data, such as from a detailed register of all healthcare professionals, would be a vital asset to policy and planning in health human resources.

## Author contribution

Study design: MPB, SN

Data collection: MPB

Data analysis: MPB

Study supervision: SN

Manuscript writing and revisions: MPB, SN
